# Dysregulation of ferroptosis may involve in the development of non‐small‐cell lung cancer in Xuanwei area

**DOI:** 10.1111/jcmm.16318

**Published:** 2021-02-02

**Authors:** Guangjian Li, Jiapeng Yang, Guangqiang Zhao, Zhenghai Shen, Kaiyun Yang, Linwei Tian, Qinghua Zhou, Ying Chen, Yunchao Huang

**Affiliations:** ^1^ Department of Thoracic Surgery I The Third Affiliated Hospital of Kunming Medical University & Yunnan Cancer Hospital Kunming China; ^2^ Shenzhen Institute of Hong Kong University Shenzhen China; ^3^ Lung Cancer Center West China Hospital Sichuan University Chengdu China

**Keywords:** ferroptosis, haptoglobin, lung cancer, thioredoxin, Xuanwei

## Abstract

The Xuanwei area of Yunnan Province, China, is one of the regions suffering from the highest occurrence and mortality rate of lung cancer in the world. Local residents tend to use bituminous coal as domestic fuel, which causes serious indoor air pollution and is established as the main carcinogen. After the local government carried out furnace and stove reform work, lung cancer rate including incidence and mortality among residents remains high. We herein wonder if there are specific mechanisms at protein level for the development of non‐small‐cell lung cancer (NSCLC) in this area. We investigated the changes of protein profiling in tumour of the patients from Xuanwei area. Tandem mass tag (TMT) was employed to screen the differential proteins between carcinoma and para‐carcinoma tissues. We identified a total of 422 differentially expressed proteins, among which 162 proteins were significantly up‐regulated and 260 were downregulated compared to para‐carcinoma tissues. Many of the differentially expressed proteins were related to extracellular matrix (ECM)‐receptor interaction, focal adhesion, PI3K/AKT pathway and ferroptosis. Further experiments on the two differential proteins, thioredoxin 2 (TXN2) and haptoglobin (HP), showed that the change of their expressions could make the lung cancer cell lines more resistant to erastin or RSL‐induced ferroptosis in vitro, and promote the growth of tumour in nude mice. In conclusion, this study revealed that aberrant regulation of ferroptosis may involve in the development of lung cancer in Xuanwei area.

## 1 INTRODUCTION

The number of deaths due to cancer accounts for about 12% of the total deaths each year worldwide, and there are over 1 000 000 new cases of cancer per year.^1^ Lung cancer has become the leading cause of cancer deaths in humans. Xuanwei area in Yunnan province, including Xuanwei, Fuyuan, Qilin and Zhanyi Counties (E 103°35′30″ to 104°40′50″, N 25°53′50″ to 26°44′50″), located in south‐western China, has a population of about 1.4 million, and its incidence of lung cancer is among the highest in the world. Especially, the incidence of lung cancer among non‐smokers is 400/100 000, which is 20 times higher than the national average.^2^, ^3^ Previous studies have associated this excessive incidence of lung cancer with the domestic combustion of ‘smoky coal’, especially burning the coals in unvented households.^4^, ^5^ The smoky coal typically means the locally obtainable Late Permian bituminous coal,^6^ which releases high level of visible smoke upon combustion. This kind of coal is available from many local coal mines within Xuanwei region and constitutes the primary fuel source for native residents. Several researches have proposed that the smoky coal combustion products like polycyclic aromatic hydrocarbons (PAHs) and air pollution fine‐grained matter such as crystalline quartz particulate are classified as mutagens to human cancer.^7^, ^8^ The Yunnan Provincial Government has carried out furnace and stove reform work in Xuanwei area, hoping to reduce the incidence of lung cancer by reducing indoor coal‐burning air pollution. However, the results of the Third National Sampling Survey of Cause of Death showed that the lung cancer mortality rate in the whole Xuanwei area increased most significantly nationwide.^9^ Therefore, there could be other more important carcinogens.

Research to date on alternative potential driver risks for prevalent lung cancer in Xuanwei has been revealed. The primary risk arises from environmental pollution. The roles of some metal elements, especially heavy metal elements, are partially known in carcinogenesis in the context of lung cancer. As studies of Yunnan soil are observed, heavy metal pollution with metals of V, Cd, Cr, Cu, Mn, Co, Ni, Pb, As and Zn exceeds the vicinities concentrations, which denotes these metals have heavily polluted the street dusts of Xuanwei. Among which, Cd, Cr, Ni, Cr and As, are considered to be carcinogenic for human with long‐term dusts exposure, and Cr is especially relevant for local children's risk.^10^ The toxicity of Pb on lungs has been found to related to the increases in Nuclear factor kappa B (NF‐κB) and aryl hydrocarbon receptor (AHR) levels, which further lead to the increases of nitric oxide synthase (iNOS) and cytochromes of the 1 subfamily (CYP1A1).^11^ Another study shows that Cr(VI) causes alterations in DNA modification and microRNAs.^12^ Moreover, LUSC patients show lower Ti expression but higher miR‐24‐3p and miR‐28‐5p.^13^ Some other studies have found the association between the high lung cancer incidence and the contamination of metal elements in Xuanwei area.^10^, ^14^ A recent study shows that the mean levels of heavy metals especially in the magnetic fractions within the road dust are rather higher than their background values.^15^

Thus, we suggested that the geological composition of the Xuanwei area may have special molecular mechanisms in the development of lung cancer in these area . Furthermore, we would analyse the protein profiling changes of the lung cancer tissues form the patients of Xuanwei area, providing more insights to the large excess incidence of lung cancer in this area and finding specific bio‐molecular mechanisms involving the regional lung cancer.

## 2 MATERIALS AND METHODS

### 2.1 Ethical statement

The experimental protocols were approved by the Ethics Committee of The Third Affiliated Hospital of Kunming Medical University and followed the guidelines of the 1975 Declaration of Helsinki. All patients in this study provided written informed consent.

### 2.2 Specimen collection

Total 20 lung cancer tissues and 20 adjacent normal lung tissues were collected from The Third Affiliated Hospital of Kunming Medical University. All subjects were born and lived in Xuanwei area for more than 3 generations and with local bituminous coal contact history over 10 years. The pathologic diagnosis was performed by a pathologist for all subjects of lung carcinoma. All the patients had not received any anti‐tumour treatment before specimen collection. The clinic pathological manifestations were acquired from the medical records of the patients. All the collected specimens were stored at −80°C until use.

### 2.3 Cell culture

All of the lung cancer cell lines, including A549 and NCI‐H1299, were purchased from Shanghai Cellular Research Institute and maintained in RPMI‐1640 medium (ScienCell) supplemented with 10% foetal bovine serum, 100 U/mL penicillin and 100 μg/mL streptomycin in a humidified incubator at 37°C with 5% CO_2_.

### 2.4 Western blotting

The cells were treated under the indicated conditions and then efficient lysed with RIPA buffer at pH 8.0 (150 mM NaCl, 50 mM Tris, 1% Triton X‐100, 0.5% sodium deoxycholate, 0.1% SDS). All the samples were quantified using Pierce^TM^ bicinchoninic acid (BCA) assay (Thermo Fisher Scientific Inc.) to ensure equal loading of proteins. The cellular protein samples were separated by SDS‐PAGE and then transferred to a polyvinylidene difluoride membrane (Millipore). After blocked by 5% BSA, the membrane was probed with the primary antibody and then incubated with HRP‐conjugated secondary antibody in TBST. The primary antibodies include anti‐TXN2 (1:1000 dilution, Abcam, ab185544) and anti‐HP (1:1000 dilution, Abcam, ab256454).

### 2.5 Quantitative RT‐PCR

RNA extraction with a kit (Qiagen) and the PCR was analysed using an ABI 7300 Real‐Time PCR System (Applied Biosystems) with the DreamTaq Green PCR Master Mix (2×). The sequences of the primer pairs used for qRT‐PCR were from previous report and presented as follows: HP HP‐Forward primer: 5′‐ATGACCTCCTTCACAAGCGC‐3′, HP‐Reverse primer: 5′‐GAAGAGCCCTCAGGCTGGACT‐3′; TXN2‐Forward: 5′‐CTGGTGGCCTGACTGTAACAC‐3′, TXN2‐Reverse: 5′‐TGACCACTCGGTCTTGAAAGT‐3′; β‐Actin‐Forward: 5′‐AGAGCTACGAGCTGCCTGAC‐3′, β‐Actin‐Reverse: 5′‐AGCACTGTGTTGGCGTAC‐3′. Relative quantitation of gene expression was assessed with analysing samples in triplicate and normalized by β‐actin level.

### 2.6 Cell viability assay

Cell viability was measured by a CCK‐8 kit (Beyotime) following the manufacturer's instruction.

### 2.7 Iron quantification

Cells were seeded with 5 × 10^6^ cells per plate and treated with erastin or RSL3 for 24 hours. The iron assay kit (ab83366) was performed according to the manufacturers' protocols. The concentration of intracellular ion was determined with a microplate reader at the absorption of 593 nm.

### 2.8 Reactive oxygen species (ROS) assay

A superoxide indicator, dihydroethidium (DHE, Invitrogen), was added at the concentration of 5 μM into the medium and incubated with the cells for 1 hour at 37°C. Fluorescence was detected following the kit instructions with the excitation at 485 nm and emission at 527 nm.

### 2.9 Lipid peroxides measurement

To visualize the lipid ROS, cells of the designate treatments were stained with 2 μmol/L C11‐BODIPY581/591 probe (Molecular Probes Inc.) in accordance with the manufacturer's instructions, 30 minutes at 37°C in the dark; the cells were then stained with nuclear stain DAPI (G1012; Servicebio) for 10 minutes. Subsequently, cells were washed and ready for the analysis of Liperfluo with measurement of the intensity of C11‐BODIPY581/591 fluorescence.

### 2.10 Malondialdehyde (MDA) assay

As a product of lipid peroxidation, MDA was measured based on the reaction between MDA and thiobarbituric acid using a commercial kit (Beyotime Biotechnology).^18^ The activity of enzyme was recorded as U/mg protein. The mean values from assays were from 3 independent experiments.

### 2.11 Cell transfection

The siHP, siTXN2, siControl, the control pcDNA, pcDNA‐TXN2 and pcDNA‐HP plasmids were synthesized by GenePharma Biotech and transfected into lung cancer cells with Lipofectamine 2000 (Bioleaf Technology Co.) according to the manufacture's instruction.

### 2.12 Animal experiments

All animal experiments strictly adhered to local regulations as well as LAWER (Laboratory Animal Welfare Ethics Review) guidelines (Andersen and Winter, 2017; Herrmann and Flecknell, 2018) and were approved by the local authorities before initiation. After 48 hours of transfection to the A549 cell line with blank vector or combined siTXN2 and HP OE, the indicated tumour cells of 1 × 10^6^ in 100 μL of phosphate buffer saline (PBS) were planted into the back of BALB/c‐nu mice. When the tumours reached 50 mm^3^ at day 2, mice (n = 24) were allocated randomly into 3 groups and treated with PBS, erastin (30 mg/kg intraperitoneally, twice every other day) or RSL (10 mg/kg intraperitoneally, twice every other day). The tumour volume (tv) of experimental mice was observed 3 times every week. The tv was determined following equation: tv = ab^2^/2, in which a means the length of the tumour, and b means the width. The tumours were separated after mice were killed.

### 2.13 High‐performance liquid chromatography (HPLC) and mass spectrometry analysis

Each sample was prepared by subjecting to trypsin digestion and TMT labelling. After that, the resulted peptides were fractionated with a high‐performance liquid chromatography (HPLC) system, and then, analysis of the peptides was performed with the Q Exactive^TM^ mass spectrometer (Thermo Finnigan).

HPLC consisted Easy nLC system with C18 column (10 cm × 75 μm i.d., 3 μm particle size) (Thermo Scientific); mobile phase A (0.1% formic acid in H_2_O) and mobile phase B (85% acetonitrile with 0.1% formic acid in H_2_O) consisted the binary solvent gradient; and the elution procedure was performed as follows: initial A/B was 100:0 in volume, whereas B was linear from 0% to 35% at the first 50 minutes, and then reached to 100% at 55 minutes and held for another 5 minutes. The flow rate of mobile phase during gradient elution was 250 nL/min. Injection with an amount of 10 μL of samples and all samples was kept in an automatic injector at 4°C during the sequential analysis. Temperature of the column was kept at constant 25°C. Quality control samples were deployed in the sample queue to surveil the reliability of the raw data.

Mass spectrometry analyses were conducted with a Q‐Exactive mass spectrometer with an orbitrap analyzer, using positive ESI. The ions with a range of 300 to 1800 m/z can be acquired. The duration of dynamic exclusion was set 40.0 seconds. The resolution for the HCD spectra of MS/MS was set to 17 500 at m/z of 200. The normalized collision energy was set at 30 eV, and the under‐fill ratio at 0.1%. Quality control samples were ran at an interval of 7 samples.

### 2.14 Data processing

Mascot (version 2.5, Matrix Science) was used to analyse the MS/MS spectra. Then, the data set was searched with the SWISS‐PROT database. The TMT peptides were quantified by Scaffold version 4.10.0 (Proteome Software, Inc.). Significant changes were defined as a threshold of ±1.2‐fold change with *P* value < .05. Gene Ontology annotation, including biological processes, cellular components and molecular functions, was performed with BLAST2GO (version 2.7), and enrichment analysis of pathways for the differentially expressed proteins employed Kyoto Encyclopedia of Genes and Genomes (KEGG) automatic annotation server.

### 2.15 Transmission electron microscopy (TEM)

The cells were washed with pre‐cooled PBS (pH 7.4) and then post‐fixed in 2.5% glutaraldehyde and 1% osmium tetroxide in phosphate‐buffered solution. The samples were sequentially sliced and stained with 2% uranyl acetate (UA), dehydrated in ethanol with graded series and embedded in an epoxy resin. Finally, the sections (70‐90 nm) were stained with UA and lead citrate. Ultrastructural images were acquired with a TEM (Hitachi HT7700).

### 2.16 Statistical analysis

Statistic Package for Social Science (SPSS) 20.0 statistical software was employed to perform data analysis. The differences were denoted with one‐way ANOVA and considered as significant at *P* < .05.

## 3 RESULTS

### 3.1 Identification of differentially expressed gene in lung cancer

**FIGURE 1:**
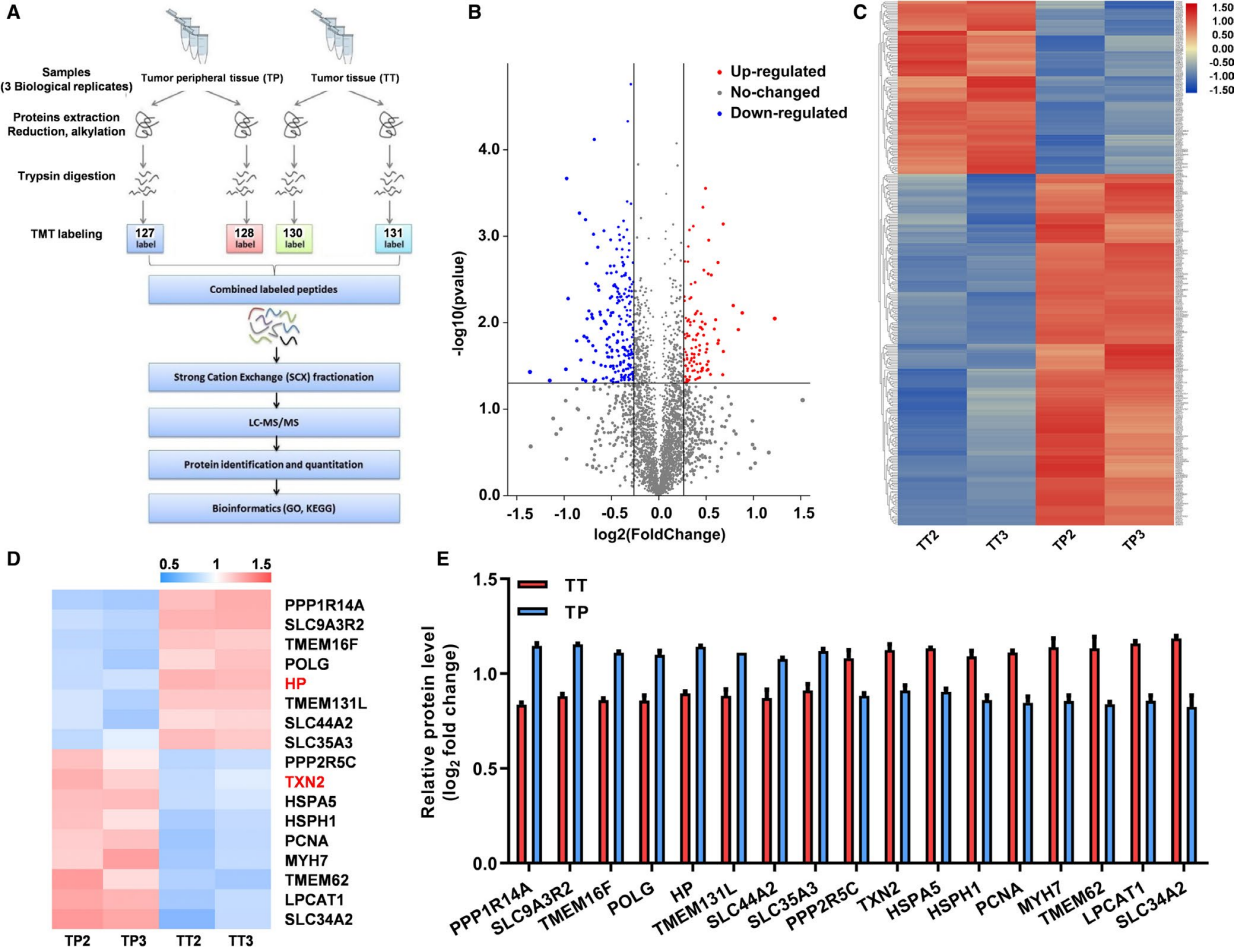
The proteomic analysis for the clinic carcinoma and para‐carcinoma tissues of the patients diagnosed as lung cancer in Xuanwei. A, The scheme of the proteomic analysis in this study. B, The volcano plot showed the differentially expressed genes in the clinic samples. C, Hierarchical clustering of the differentially expressed genes. For hierarchical clustering, blue and red indicate decreased and increased expression, respectively. The proteins were clustered by hierarchical clustering using the complete linkage algorithm and Pearson correlation metric in R. D, Heatmap of the proteins potentially related to ferroptosis. E, Relative protein level of the molecules potentially related to ferroptosis; data were collected from the proteomic analysis results

The scheme of the proteomic analysis is presented in Figure 1A. A total of 11 134 peptides in the clinic tissue samples were detected, which covers 3473 proteins; of these, 422 differentially expressed proteins (fold‐change >1.2 or <0.83 in comparison with the para‐carcinoma tissues, *P* < .05) were found in the analysis with quantitative information and were included in the next bioinformatics analysis. A volcano chart was drawn according to 2 factors, the fold‐change and the p value obtained by t test, to show the significant difference in data between the two groups of samples (Figure 1B). We used the hierarchical cluster to compare the differentially expressed proteins of the representative cases, showing with the heat map (Figure 1C). The chart indicated that the threshold of fold‐change set in the current study can effectively separate the cancer and the para‐carcinoma groups, and the data of each case from the 2 groups were reproducible. According to the description about in GO terms and several recently published studies, various proteins, including TXN2, HP, PCNA, MYH7, POLG, TMEM62, TMEM16F, TMEM131L, SLC34A2, SLC35A3, SLC9A3R2, SLC44A2, HSPH1, HSPA5, LPCAT1, PPP1R14A and PPP2R5C, were screened out (Figure 1D) and subsequently subjected to qPCR validation (Figure 1E).

### 3.2 OncoPPi network and critical pathway hubs

**FIGURE 2:**
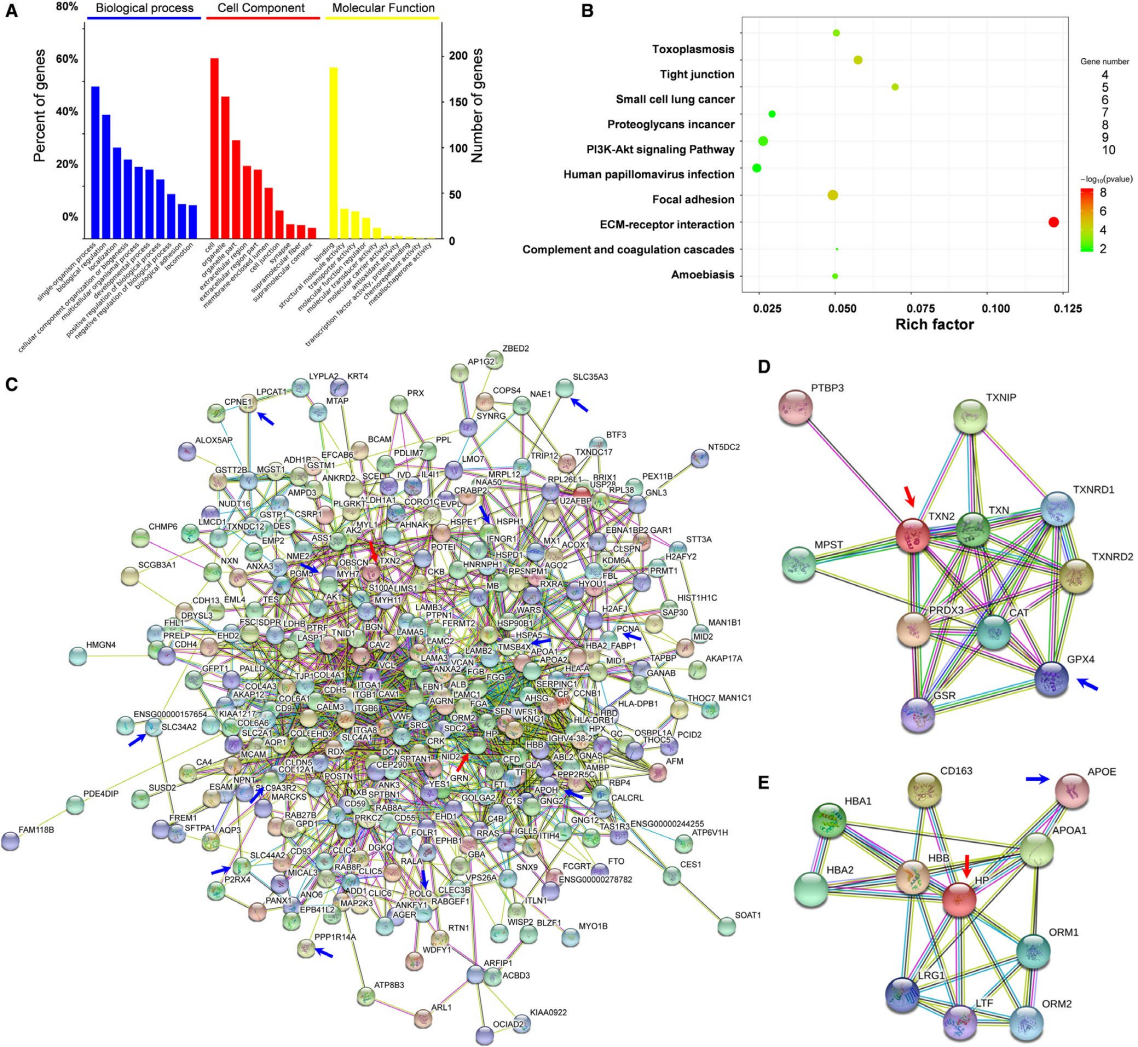
Bioinformatic analysis for the proteomic data and protein‐protein interaction (PPI) networks. A, GO classification of the differential proteins by biological process, cellular component and molecular function. B, KEGG analysis of the differential proteins. To the left of each plot: KEGG terms. Under each plot: the percentage of the sequence. C, PPI network of all DEPs with a log2 (fold change) >1.2 or <0.83. The data were uploaded to the STRING 11.0 software to analyse the interactions among all DEPs. D, PPI network of TXN2‐related proteins, including NDEPs and DEPs. E, PPI network of TXN2‐related proteins, including NDEPs and DEPs. Coloured ball: the changed protein; yellow line: text mining; purple line: experiments; blue line: databases; light blue: homology; black line: co‐expression; green line: neighbourhood; red line, gene fusion; deep blue: co‐occurrence. DEPs: differentially expressed proteins; NDEPs: non‐differentially expressed proteins

The 422 proteins (162 up‐regulated and 260 down‐regulated versus para‐carcinoma tissues) were annotated according to their biological process, cellular component and molecular function by BLAST2TO (Figure 2A). Biological processes analysis showed that these proteins were mainly involved in single‐organism process, localization, cellular component organization or biogenesis, biological regulation and multicellular organismal process. Cellular component analysis showed that most of the differential proteins were located in the extracellular region, membrane‐enclosed lumen and organelle. Molecular function analysis revealed that a large proportion of these proteins played a role in molecular function regulator, structural molecule activity, protein binding, transporter activity and molecular transducer activity. Pathway annotation by KEGG analysis demonstrated that these differential proteins are mainly involved in ECM‐receptor interaction, focal adhesion, small cell lung cancer, PI3K/AKT pathway and amoebiasis (Figure 2B). The above pathways might be involved in the lung cancer in this area.

TXN2 and HP were relatively in the centre of the PPI network of all differentially expressed gene (DEGs), implying some important roles that they might play in the progression of NSCLC (red arrow, Figure 2C). Other various ferroptosis‐related proteins that were indicated with blue arrows (Figure 2C) were scattered on the network, further suggesting that ferroptosis probably involved in the progression of NSCLC. In addition, we picked out TXN2 and HP to analyse more potential association with other possible molecules in ferroptosis (Figure 2D,E), and we noticed GPX4 (blue arrow), which is a documented key negative regulator of ferroptosis, and apolipoprotein E (APOE) (blue arrow), a well‐known lipid peroxidation inhibitor, are closely implicated. In addition, we noticed that there are few studies about these two proteins TXN2 and HP in ferroptosis. Based on these findings, we decided to pay more attention on these two molecules in the following research.

### 3.3 Altered expression of Txn2 and HP in lung cancer was associated with ferroptosis

**FIGURE 3:**
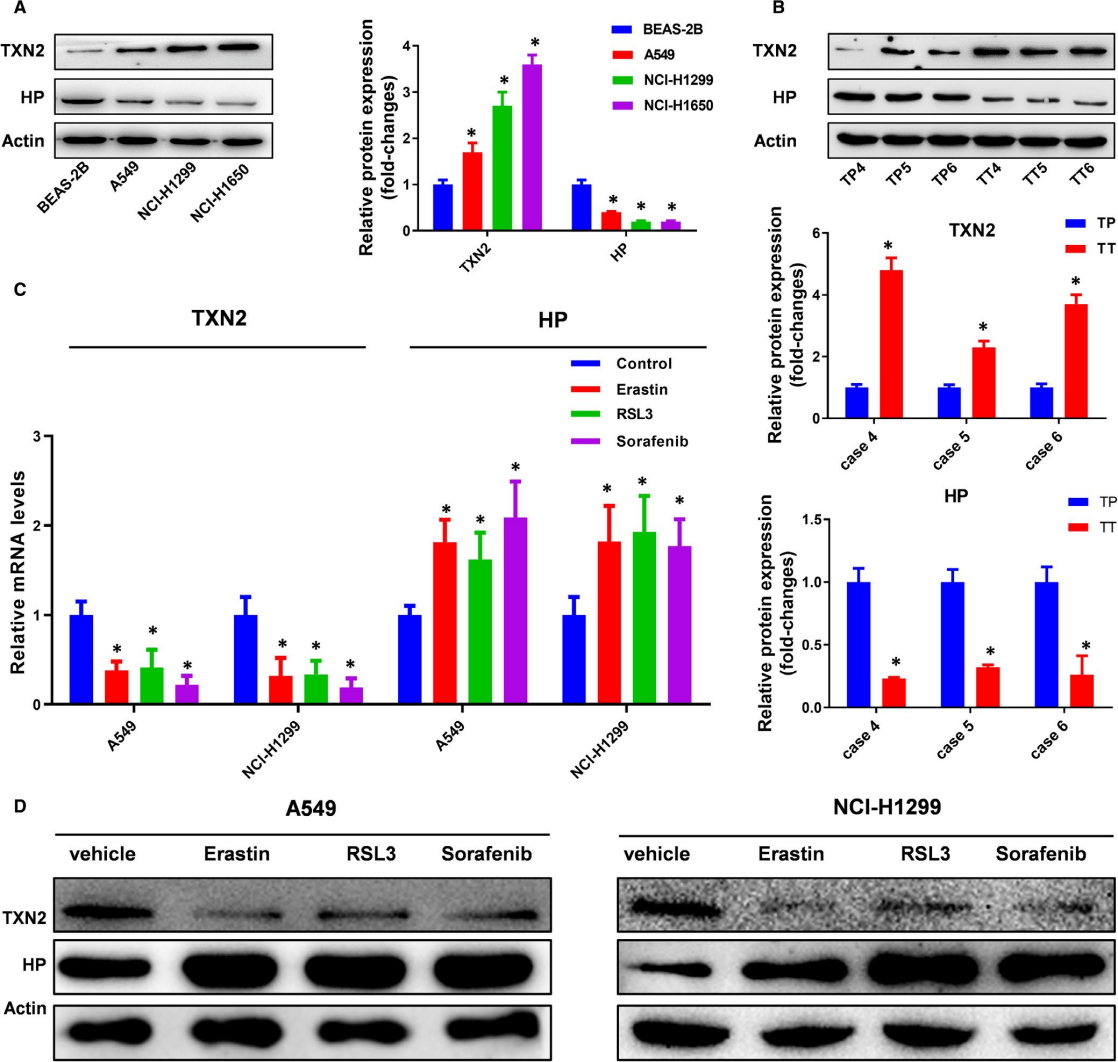
Validation of proteomics results. A, Two differentially expressed proteins TXN2 and HP in lung cell lines were detected by Western blotting. *, *P* < .05. B, The proteomics results of TXN2 and HP were validated in the clinic samples. TP, tumour peripheral tissue; TT, tumour tissue. *, *P* < .05. C, The expression of TXN2 and HP at mRNA level under the treatment of ferroptosis inducer, erastin (10 µM), RSL (1 µM) or sorafenib (5 μM). *, *P* < .05; **, *P* < .01. D, The expression of TXN2 and HP at protein level under the treatment of ferroptosis inducer, erastin (10 µM), RSL (1 µM) or sorafenib (5 μM)

We validated the changes of TXN2 and HP in proteomics using several lung cancer cell lines and clinic samples (Figure 3A,B). We observed that TXN2 was significantly decreased but HP increased in the cells treated with the ferroptosis inducers erastin, RSL or sorafenib at both mRNA and protein levels (Figure 3C,D), which indicated that the trend in proteomic analysis was reversed by the inducer. We hypothesized ‐ that regulating the levels of TXN2 and HP in lung cancer cells would influence the cell proneness to ferroptosis.

### 3.4 Survival analysis of TXN2

**FIGURE 4:**
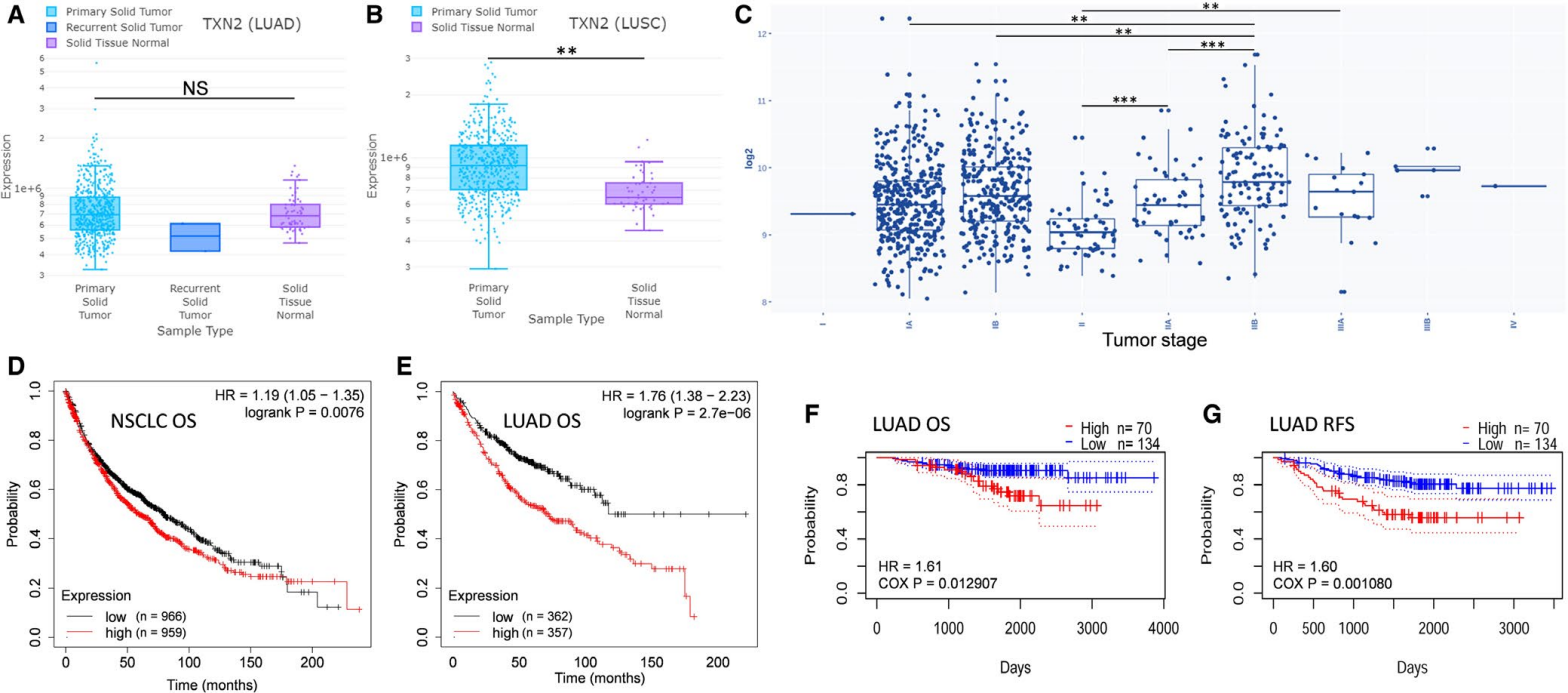
TXN2 expression profile in NSCLC and its role in prognosis. A‐B, TXN2 expression in LUAD (lung adenocarcinoma), LUSC (lung squamous cell carcinoma) and lung normal tissues. C, TXN2 expression in NSCLC, including LUAD, LUSC and LCLC (large cell lung cancer), from GEO public repository showed higher levels in at later clinical stages. D‐E, Overall survival analysis of low‐TXN2 and high‐TXN2 NSCLC (LUAD and LUSC); D) patients or LUAD patients (E); patients split by median; probe: 209078_s_at. (F‐G) Overall survival and RFS (relapse free survival) analyses of low‐TXN2 and high‐TXN2 LUAD patients; probe: 209077_at

Using the online tool DriverDBv3 (http://driverdb.tms.cmu.edu.tw/), whose data source is TCGA, TXN2 expression was found enhanced in LUSC (lung squamous cell carcinoma) but not in LUAD (lung adenocarcinoma), compared to lung normal tissue (Figure 4A,B). To further explore the possible role of TXN2 in tumour progression of NSCLC, TXN2 expression in NSCLC from GEO public repository was analysed with GENT2 (http://gent2.appex.kr/gent2/). As a result, we found higher TXN2 levels at later clinical stages, though it was lower in at some stages (Figure 4C). If overexpressed TXN2 plays a role in progression of NSCLC, the patients with different TXN2 level may have different survival results. According to the survival analysis results from Kaplan‐Meier plotter (http://kmplot.com/analysis/), the overall survival (OS) signature (high risk vs. low risk) of TXN2 (Affymetrix ID: 209078_s_at) presents high‐TXN2‐expression patients’ overall survival time is shorter than that of low‐expression NSCLC patients (hazard ratio [HR] 1.19; *P* = .0076), especially for LUAD (HR = 1.76, *P* = 2.7^e^‐6) (Figure 4D,E). In one of the above data sets (GSE31210, analysed with PrognoScan, http://dna00.bio.kyutech.ac.jp/PrognoScan/index.html), apart from the OS result in Figure 4F (PROBE ID: 209077_at) which was consistent with that in Figure 4D, high‐TXN2‐expression patients also exhibited lower RFS (relapse free survival) in Figure 4G (PROBE ID: 209077_at). But for LUSC, no significantly differences were found between low‐TXN2 and high‐TXN2 groups (data not shown).

### 3.5 Survival analysis of HP

**FIGURE 5:**
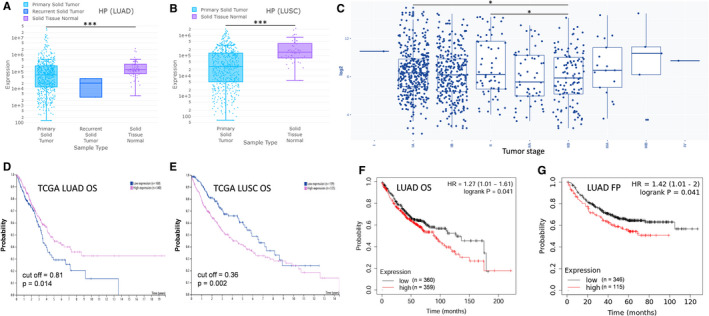
HP expression profile in NSCLC and its role in prognosis. A‐B, HP expression in LUAD, LUSC and lung normal tissues. C, HP expression in NSCLC, including LUAD, LUSC and LCLC, from GEO public repository showed higher levels in at later clinical stages. D‐E, Overall survival analysis of low‐HP and high‐HP LUAD (D) or LUSC (E) patients. F‐G, Overall survival and FP (first progression) analyses of low‐HP and high‐HP LUAD patients; probe: 206697_s_at

HP expression data in LUAD and LUSC from TCGA were analysed with online tool DriverDBv3 (http://driverdb.tms.cmu.edu.tw/), and it was found that HP was notably downregulated in both LUAD and LUSC tissues, compared to lung normal tissue (Figure 5A,B). The possible role of HP in tumour progression of NSCLC was revealed through analysis of its expression in NSCLC using GENT2 (http://gent2.appex.kr/gent2/) based on GEO public repository. However, lower HP at later clinical stages was not frequently observed (Figure 5C). According to the survival analysis results from The Human Protein Atlas (https://www.proteinatlas.org/), low‐HP‐expression patients have a poorer overall survival in LUAD (Figure 5D) (cut‐off = 0.81, *P* = .014), but quite in reverse, low‐HP‐expression patients have a better prognosis for LUSC (Figure 5E) (cut‐off = 0.36, *P* = .002). To further validate the role of HP in prognosis of NSCLC, we analysed the survival of patients with low or high HP expression using Kaplan‐Meier plotter (http://kmplot.com/analysis/) that mainly contains GEO (gene expression omnibus) data sets. Against our prediction and the aforesaid result from TCGA, the low‐HP‐expression (Affymetrix ID: 206697_s_at) patients’ overall survival was better, consistent with which, these patients showed longer time till first progression (Figure 5F,G). In addition, according the survival analysis results from GEO data sets, HP expression level showed no effect on LUSC patients’ survival (data not shown).

### 3.6 Altered TXN2 and HP linked to ferroptosis in lung cancer cell lines in vitro

**FIGURE 6:**
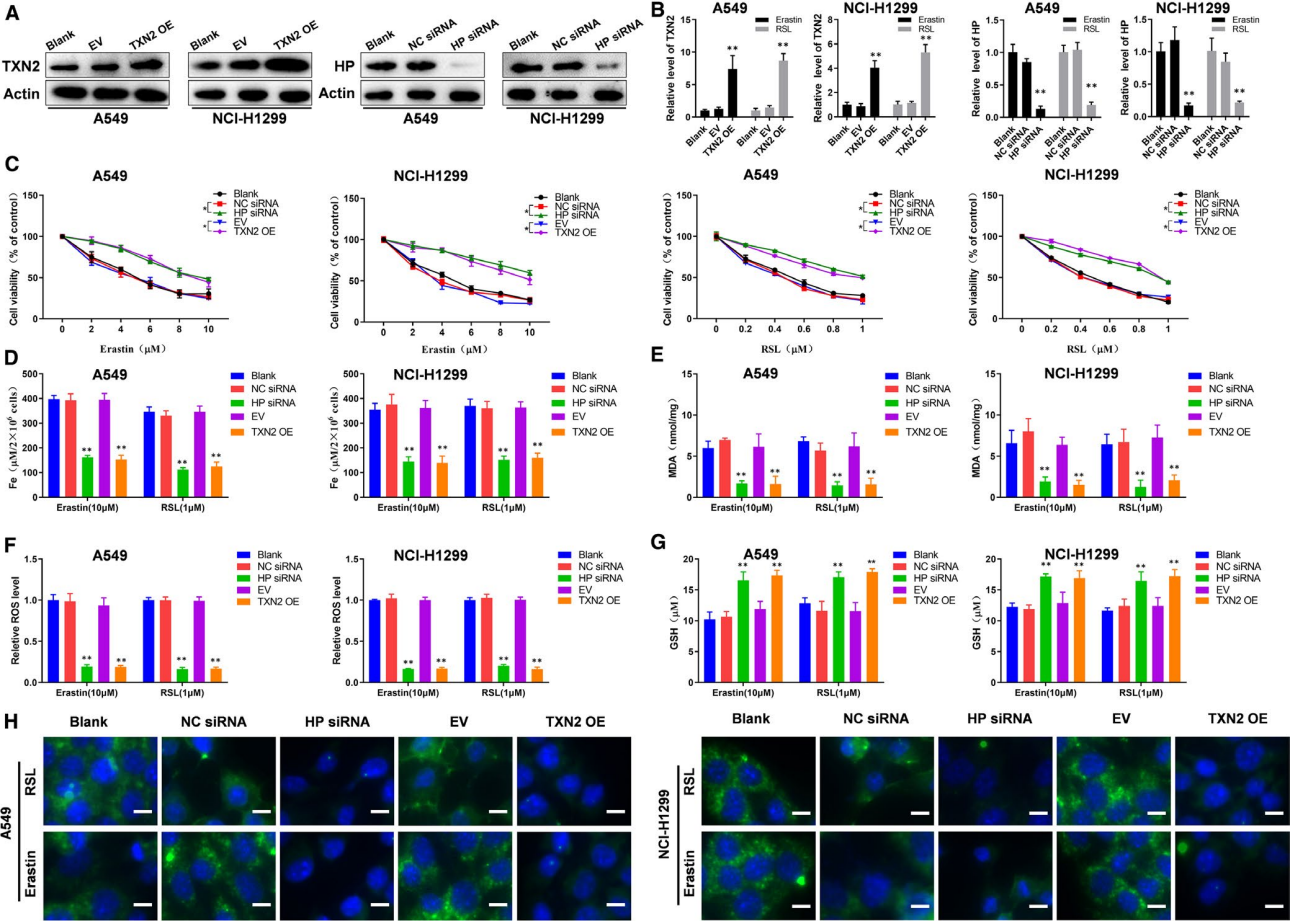
TXN2 overexpression and HP depletion promoted resistance of lung cancer cell lines through attenuating ferroptosis. A, The efficiency of transfected cells was analysed via Western blot assays. B, qPCR showed TXN2 and HP expression with erastin (10 µM) or RSL (1 µM) treatment for 24 h. C, Cell viability assays for the cells with the increasing erastin or RSL treatments for 72 h. The iron concentrations (D), MDA concentrations (E), the relative ROS levels (F) and the GSH concentrations (G) of the cells under the treatment of erastin (10 µM) or RSL (1 µM) for 24 h. H, Analysis of C11‐BODIPY581/591 fluorescence (scale bar = 10 μm). *P < .05; **, *P* < .01

We overexpressed TXN2 or interfered with HP in lung cancer cell lines (A549 and NCI‐H11299). The efficiency of TXN2 overexpression and HP depletion was confirmed by Western blotting (Figure 6A); the mRNA level of TXN2 overexpression and HP depletion induced by erastin or RSL was also detected, as shown in Figure 6B; and erastin or RSL were unable to eliminate TXN2 expression with TXN2 overexpression, whereas erastin or RSL were unable to induce HP expression with HP depletion. Cell viability results revealed that the transfected cells became more resistance to increasing dose of erastin or RSL‐induced cell death compared to non‐transfected cells by showing higher rates of survival (Figure 6C). The levels of Fe^2+^ were remarkably decreased (Figure 6D). MDA assays indicated the lower oxidative metabolism of lipids in the blank lung cancer cells under the erastin or RSL treatment, whereas the transfected cells had a lower level of membrane lipid oxidation (Figure 6E). Similarly, changes in ROS also indicated a significant decrease in oxidation levels of the transfected cells under erastin or RSL treatment (Figure 6F). The GSH assays showed that the GSH in the transfected cells was higher (Figure 6G). TXN2 overexpression or HP depletion decreased erastin and RSL‐induced lipid oxidation in lung cancer cell lines, which were shown by the green fluorescence intensity (levels of oxidized C11 BODIPY 581/591) (Figure 6H). We found in (Figure S1); however, cell deaths and MDA levels induced by ferroptosis inducers would not be reversed by caspase pharmacological inhibitor ZV AD‐FMK and the necroptosis inhibitor, Necrosulfonamide, in A549 and NCI‐H1299 cell lines, which could be inhibited by specific ferroptosis inhibitor ferrostatin. This verified the effects of ferroptosis on lung cancer cells and excluded other regulated cell death forms, like apoptosis and necroptosis. All these results suggested that TXN2 overexpression and HP depletion promoted lung cancer resistance were through attenuating ferroptosis.

**FIGURE 7:**
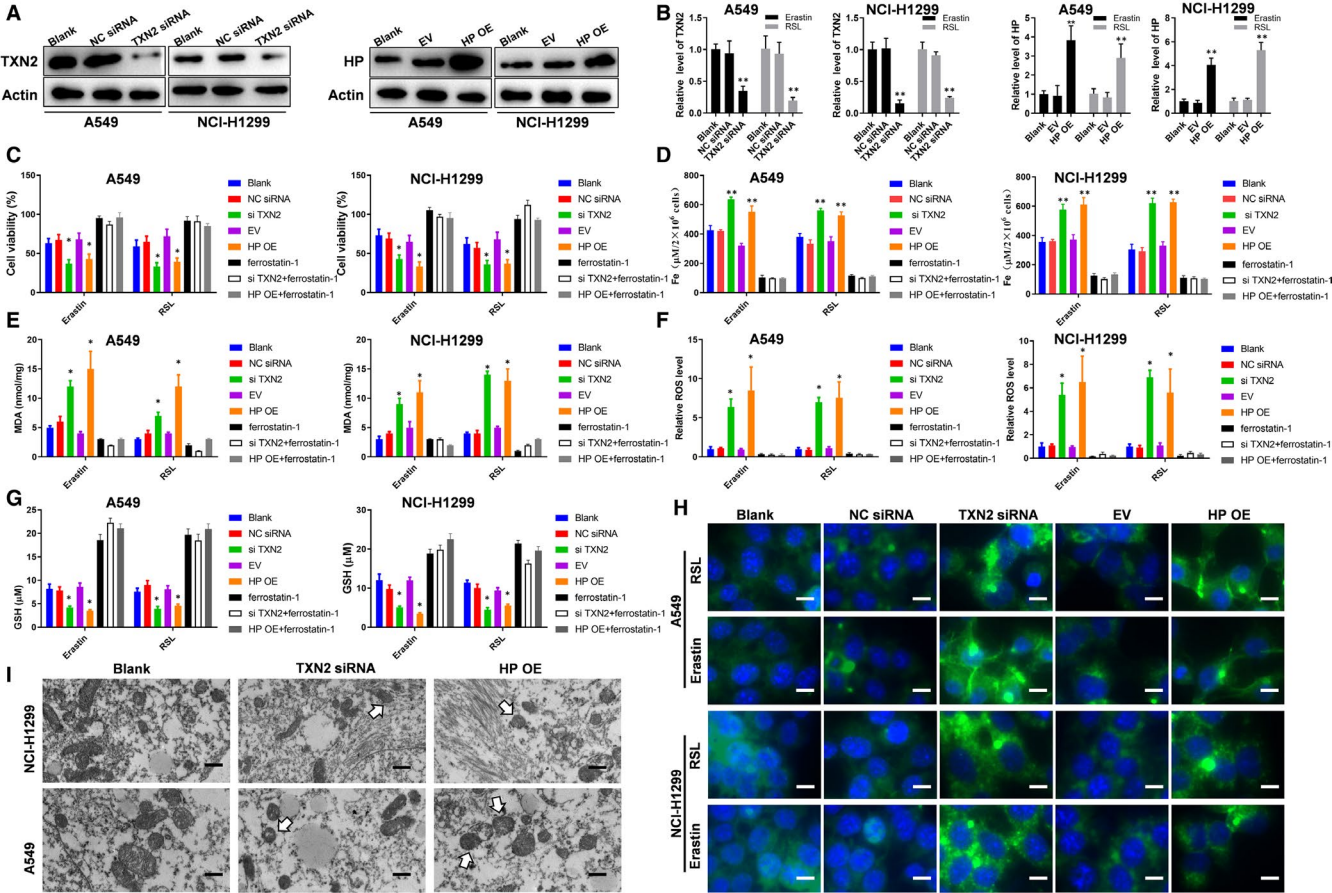
TXN2 depletion and HP overexpression enhanced ferroptosis in lung cancer cell lines A549 and NCI‐H1299. A, The efficiency of transfected cells was analysed via Western blot assays. B, qPCR showed TXN2 and HP expression with erastin (10 µM) or RSL (1 µM) treatment for 24 h. C, Cell viability assays for the cells with erastin (10 µM) or RSL (1 µM) treatment for 24 h. The iron concentrations (D), MDA concentrations (E), the relative ROS levels (F), the GSH concentrations (G) of the cells under the treatment of erastin (10 µM) or RSL (1 µM) for 24 h. H, Analysis of C11‐BODIPY581/591 fluorescence (scale bar = 10 μm). I, The alterations of mitochondrial ultrastructure of the indicated cells and white arrows indicated mitochondria (scale bar = 500 nm). NC, negative control; EV, empty vector; OE, overexpressing; *, *P* < .05, **, *P* < .01

Then, we overexpressed HP or interfered with TXN2 in lung cancer cell lines; the protein level of HP and TXN2 was detected (Figure 7A); the mRNA level of HP overexpression and TXN2 depletion induced by erastin or RSL was also detected (Figure 7B); and results showed that erastin or RSL further eliminated TXN2 expression with TXN2 knockdown, whereas erastin or RSL further induced HP expression with HP overexpression. We observed that the transfected cells became more prone to erastin or RSL‐induced ferroptosis compared to non‐transfected cells by showing lower rates of survival (Figure 7C). Moreover, we also found that the levels of Fe^2+^ (Figure 7D), MDA (Figure 7E), intracellular lipid ROS (Figure 7F) and lipid peroxidation (Figure 7H) in A549 and NCI‐H11299 were remarkably increased and GSH was decreased (Figure 7G). Nonetheless, the effect of both erastin and RSL along with HP overexpression or TXN2 depletion on cell survival levels of Fe^2+^, MDA, intracellular lipid ROS, lipid peroxidation and GSH could be reversed by ferrostatin (Figure 7C‐G).Observation by TEM revealed that the lung cancer cells with interference of TXN2 or overexpression of HP had smaller mitochondria and a decreased number of mitochondrial cristae under the treatment of erastin or RSL, and the proportion of mitochondria with ruptured outer membrane increased in these cells (Figure 7I). These results suggested that upregulating of HP but downregulating TXN2 can increase the ferroptosis rate of lung cancer cells.

### 3.7 TXN2 and HP were associated with lung cancer development in vivo

**FIGURE 8:**
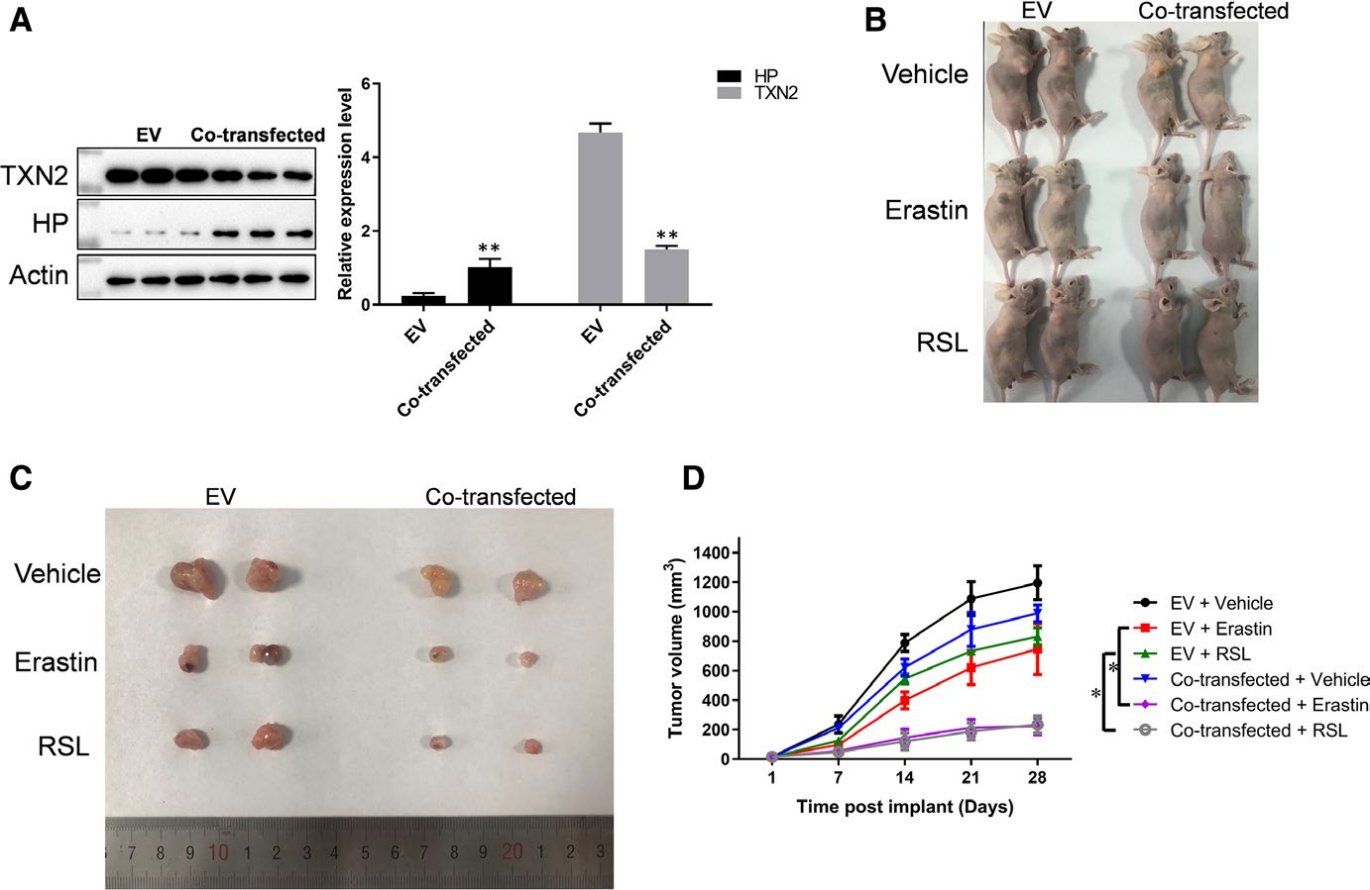
The expressions of TXN2 and HP affected ferroptosis in nude mice. A, The efficiency of TXN2 and HP. B, The tumour volume was monitored weekly. *, *P* < .05. C‐D, The tumour sizes at 28 days after implantation

In vitro, both knocking down of TXN2 and overexpression of HP were observed to modulate sensitivity of lung cancer cell lines to erastin and RSL. Afterwards, we constructed the siTXN2 but HP‐overexpressing A549 cells and performed the tumorigenicity tests using the wild‐type A549 and the co‐transfected A549 cells on nude mice. The efficiency of transfected cells is shown in Figure 8A. When the tumours reached 50 mm^3^ at day 2, mice were treated with erastin for 26 days (Figure 8B). We also found that the co‐transfected cells were more sensitive to erastin or RSL treatment in vivo, which showed that the tumour volume was much smaller than the group of wild‐type A549 at 28 days after implantation (Figure 8C,D). Therefore, promoting the development of lung cancer by inhibiting ferroptosis may be a potential and special mechanism for the high incidence of lung cancer in Xuanwei.

## 4 DISCUSSION

The Xuanwei area in Yunnan Province is a high‐risk area for lung cancer. As it is the main coal mining area in south‐western China, several geochemical studies have shown that mineral elements are associated with a high incidence of lung cancer in this area.^10^, ^16^ To date, the cause of high occurrence of lung cancer in Xuanwei area remains far from clear.

To identify the vital candidates for lung cancer pathogenesis of patients from Xuanwei, bioinformatic methods were applied firstly. Microarray study was used to analyse the altered protein profiling between carcinoma and para‐carcinoma tissues from Xuanwei area, and then, pathway enrichment was used to explore the interactions of these differential proteins and the present study showed pathways enriched in 5 processes including small cell lung cancer, ECM‐receptor interaction, focal adhesion, PI3K/AKT and amoebiasis. Apart from amoebiasis, the above pathways like ECM‐receptor interaction and adhesive attraction^17^ and PI3K/AKT^18^ are greatly noted in lung cancer previously, which may be responsible for lung cancer cell invasion and metastasis. In addition, centrally involved TXN2 and HP as two key factors of the PPI network were identified. Also, our network analysis implicated that TXN2 associated with the established negative ferroptosis regulator GPX4 and HP interconnected with the lipid peroxidation inhibitor APOE; moreover, both TXN2 and HP are ferroptosis‐related molecules. Ferroptosis is a recently identified form of cell death, which differs from apoptosis, cell necrosis and autophagy. It is caused by the accumulation of the products of iron‐dependent lipid peroxidation.^19^, ^20^ Considering the growth of cancer cell is iron dependency, which means cancer cell including lung cancer can evade apoptosis but to be more susceptible to an iron‐induced ferroptosis. Morphologically, the cells showed a decrease in volume, an increase in mitochondrial membrane density and disappearance of mitochondrial cristae during ferroptosis.^21^ At the molecular level, phospholipid‐bound polyunsaturated fatty acids (PL‐PUFAs) are peroxidized during ferroptosis to generation ROS and achieve cell kill, which can be eliminated by 3 parallel pathways: GPX4‐glutathione, fibroblast‐specific protein 1 (FSP1)‐CoQ10 and guanosine triphosphate cyclohydrolase 1 (GCH)1‐BH4.^22^ Several other molecules have been found to regulate ferroptosis in lung cancer, such as the erythroid 2‐related factor 2 (Nrf2)^23^ and serine threonine tyrosine kinase 1 (STYK1).^24^ Besides, growing evidences suggest an important lethal role for iron and ferroptosis in lung cancer.^25^ Given that the main emitted chemical particle from coal combustion is iron in Xuanwei, which is reducible and could generate ROS, a study focused on the valence state of iron in size‐resolved particle found that oxidizable iron accounts for a large proportion of raw coal samples, whereas a large percentage of redox‐active fractions of iron (46%‐78%) in fly ash particles after coal combustion. In addition, the concentration of ·OH was higher in fine particles than coarse particles.^26^ Thus, we guess ion may link to lung cancer, and ferroptosis may be responsible for Xuanwei lung cancer.

As an important antioxidant defence mechanism, TXN system has attracted researchers' attention to its role in ferroptosis.^27^ The persistent activation of antioxidant systems and overexpression of thioredoxin via genetic alterations in Nrf2 and Keap1 also contributes to carcinogenesis.^28^ Moreover, a recent study reported genetic knockdown of TXN leads to accumulation of lipid ROS levels and induces an antioxidant response to cause ferroptosis, a potential pro‐ferroptotic agent due to its inhibition of TXN would be an anti‐cancer therapeutic strategy.^29^ HP is a plasma protein that has the ability to combine with free haemoglobin, which allows hepatic recycling of heme iron to function in preventing kidney from damage. HP also has antibacterial activity and plays an essential role in regulating responses under the acute phase.^30^ The expression of HP was found increased after intracerebral haemorrhage in vivo, which could prevent haemoglobin‐induced neuronal ferroptosis is as an antioxidant.^31^ HP polymorphism causes change in haemoglobin‐bind capacity, antioxidant and iron‐recycling activities, which may be important for effective oxidative stress response in lung cancer pathogenic process.^32^ Specifically, in the present study, we supposed that HP and TXN2 may be involved in ferroptosis and contribute to lung cancer in Xuanwei area.

It was revealed that TXN2 up‐regulated and HP downregulated in lung cancer cell tissues of Xuanwei patients and 3 lung cancer cell lines. Public data also supported that TXN2 enhanced in LUSC but not in LUAD, HP downregulated in both LUAD and LUSC. Online data showed higher TXN2 levels at later clinical stages, whereas lower HP at later clinical stages was not frequently observed. Kaplan‐Meier survival analysis suggested that higher TXN2 levels and lower HP levels might be unfavourable factors of in LUAD prognosis.

In addition, the results also indicated that ferroptosis inducer erastin or RSL downregulated TXN2 and up‐regulated HP. Further experiments with overexpressed TXN2 or interfered with HP in lung cancer cell lines demonstrated that TXN2 overexpression and HP depletion promoted lung cancer resistance to erastin or RSL‐induced cell death were through attenuating ferroptosis, whereas upregulating of HP but downregulating TXN2 increased the ferroptosis rate of lung cancer cells. The increase of cell death and MDA by erastin and RSL could not be affected with inhibitors of these two pathways in our study, which distinguished ferroptosis from apoptosis and necroptosis. That ferrostatin inhibited the inhibitory effects of altered TXN2 and HP may validate functions of TXN2 and HP during ferroptosis. Besides, co‐transfected with siTXN2 and HP overexpression vector significantly increased the size, volume of the tumour in vivo. Together, our results demonstrate that down‐regulated TXN2 and up‐regulated HP made the cancer cells more resistant, which could be a specific mechanism for the lung cancer in Xuanwei area. Further studies are needed to verify the changes in a larger cohort and to establish the association between the molecules and the high incidence of lung cancer in Xuanwei area.

## CONFLICT OF INTEREST

The authors confirm that there are no conflicts of interest.

## AUTHOR CONTRIBUTION

Guangjian Li: Data curation (equal); Formal analysis (equal); Methodology (equal); Resources (equal); Writing‐original draft (equal). Jiapeng Yang: Data curation (equal); Formal analysis (equal); Methodology (equal); Resources (equal); Writing‐original draft (equal). Guangqiang Zhao: Formal analysis (equal); Resources (lead); Visualization (lead). Zhenghai Shen: Formal analysis (equal); Resources (equal); Software (equal); Visualization (equal). Kaiyun Yang: Formal analysis (equal); Resources (equal); Software (equal); Visualization (equal); Writing‐review & editing (equal). Linwei Tian: Formal analysis (equal); Resources (equal); Software (equal); Visualization (equal); Writing‐review & editing (equal). Qinghua Zhou: Formal analysis (equal); Resources (equal); Software (equal); Visualization (equal). Ying Chen: Conceptualization (equal); Funding acquisition (equal); Project administration (equal); Supervision (equal); Writing‐review & editing (equal). Yunchao Huang: Conceptualization (equal); Funding acquisition (equal); Investigation (equal); Project administration (equal); Supervision (equal); Validation (equal); Writing‐review & editing (equal).

## Supporting information

Figure S1

## Data Availability

The data that support the findings of this study are included in the manuscript and supplementary materials.
