# Corrigendum

**DOI:** 10.1111/jcmm.17075

**Published:** 2022-01-08

In Guangjian Li et al.,^1^ an incorrect image was used for panel A549/HP OE in Figure 7I. The correct figure is shown below. The authors confirm all results and conclusions of this article remain unchanged.

**FIGURE 7:**
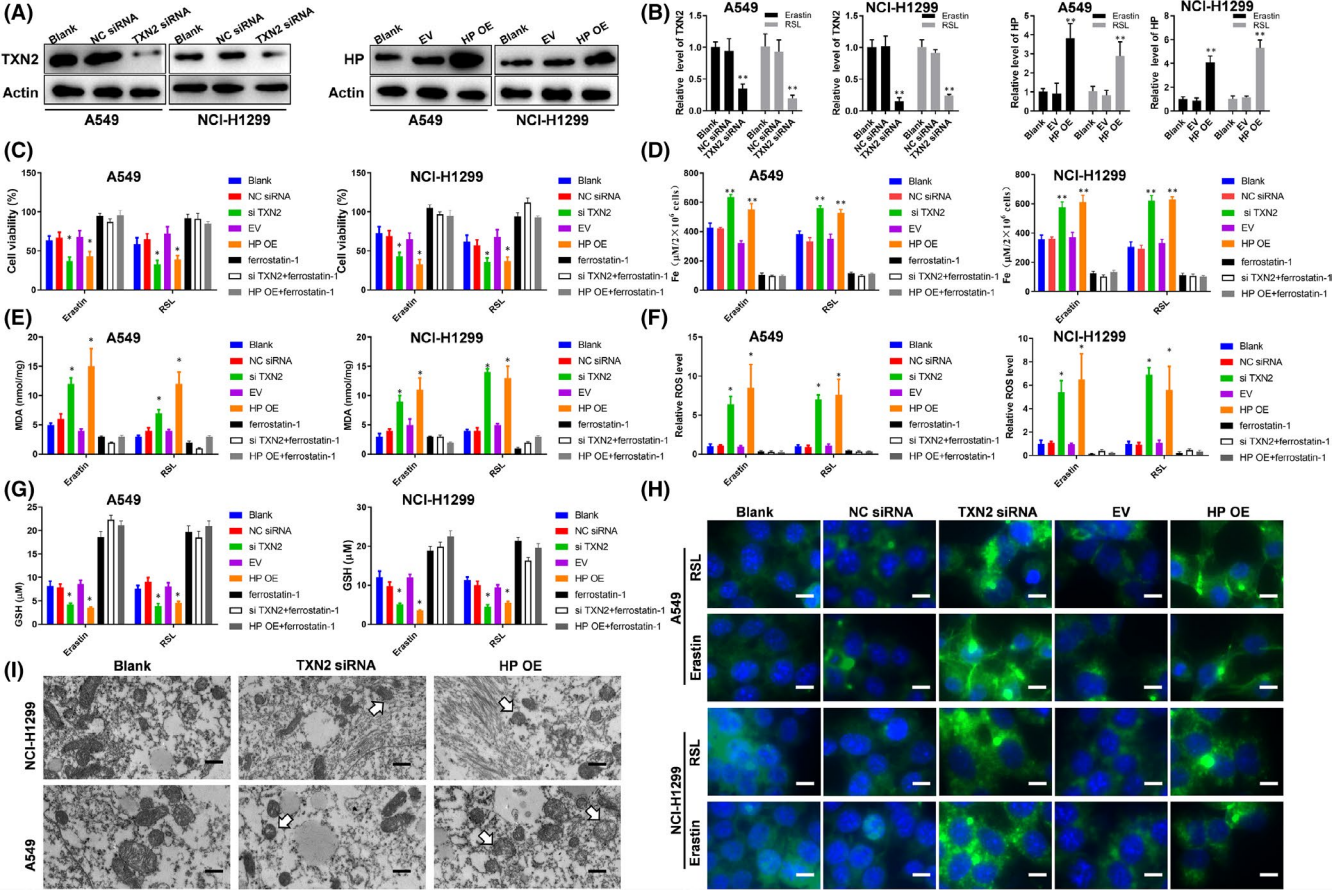
TXN2 depletion and HP overexpression enhanced ferroptosis in lung cancer cell lines A549 and NCI‐H1299. (A) The efficiency of transfected cells was analysed via Western blot assays. (B) qPCR showed TXN2 and HP expression with erastin (10 µM) or RSL (1 µM) treatment for 24 h. (C) Cell viability assays for the cells with erastin (10 µM) or RSL (1 µM) treatment for 24 h. The iron concentrations (D), MDA concentrations (E), the relative ROS levels (F), the GSH concentrations (G) of the cells under the treatment of erastin (10 µM) or RSL (1 µM) for 24 h. H, Analysis of C11‐BODIPY581/591 fluorescence (scale bar =10 μm). (I) The alterations of mitochondrial ultrastructure of the indicated cells and white arrows indicated mitochondria (scale bar =500 nm). NC, negative control; EV, empty vector; OE, overexpressing; *p < .05, **p < .01
