# Supplementary material for: Dysregulation of ferroptosis may involve in the development of non‐small‐cell lung cancer in Xuanwei area
Source: J Cell Mol Med. 2021 Feb 2;25(6):2872–84. doi: 10.1111/jcmm.16318 (PMC7957160; doi:10.1111/jcmm.16318)
Supplement: Supplementary file 1 — Figure S1 [file JCMM-25-2872-s001.docx]

**Figure S1.** The effects of several ferroptosis inducers on lung cancer cell lines. The cells were induced ferroptotic by the agent of erastin (10 μM), RSL (1 μM) or sorafenib (5 μM), showing a decreased cell viability and increased MDA level, and these effects can be reversed by ferroptosis inhibitor ferrostatin-1 at 1 μM. *, p < 0.05.
